# Valorization of Second Cheese Whey Through Microalgae-Based Treatments: Advantages, Limits, and Opportunities

**DOI:** 10.3390/biotech14040079

**Published:** 2025-10-09

**Authors:** Gloria Sciuto, Nunziatina Russo, Cinzia L. Randazzo, Cinzia Caggia

**Affiliations:** 1 Department of Agriculture, Food and Environment (Di3A), University of Catania, Via S. Sofia 100, 95123 Catania, Italy; gloria.sciuto@phd.unict.it (G.S.); nunziatinarusso83@gmail.com (N.R.); cinzia.randazzo@unict.it (C.L.R.); 2 ProBioEtna SRL, Spin Off at the University of Catania, Via S. Sofia 100, 95123 Catania, Italy

**Keywords:** dairy wastewater, biotechnological approaches, biomass production, sustainable processes, compound recovery, circular economy

## Abstract

The dairy sector produces considerable amounts of nutrient-rich effluents, which are frequently undervalued as simple by-products or waste. In particular, Second Cheese Whey (SCW), also known as scotta, exhausted whey, or deproteinized whey, represents the liquid fraction from ricotta cheese production. Despite its abundance and high organic and saline content, SCW is often improperly discharged into terrestrial and aquatic ecosystems, causing both environmental impact and resource waste. The available purification methods are expensive for dairy companies, and, at best, SCW is reused as feed or fertilizer. In recent years, increasing awareness of sustainability and circular economy principles has increased interest in the valorization of SCW. Biological treatment of SCW using microalgae represents an attractive strategy, as it simultaneously reduces the organic load and converts waste into algal biomass. This biomass can be further valorized as a source of proteins, pigments, and bioactive compounds with industrial relevance, supporting applications in food, nutraceuticals, biofuels, and cosmetics. This review, starting from analyzing the characteristics, production volumes, and environmental issues associated with SCW, focused on the potential of microalgae application for their valorization. In addition, the broader regulatory and sustainability aspects related to biomass utilization and treated SCW are considered, highlighting both the promises and limitations of microalgae-based strategies by integrating technological prospects with policy considerations.

## 1. Introduction

The dairy sector produces large amounts of liquid by-products, often characterized by high organic load and varying composition depending on the product type, production method, and location [1]. Among these streams, cheese whey (CW) represents a major fraction. Second cheese whey (SCW, also known as scotta) has an even greater impact in terms of volume and environmental burden. SCW, generated during whey cheese production, accounts for more than 90% of the original volume of whey and presents high biochemical oxygen demand (BOD) and chemical oxygen demand (COD) [2,3]. These high nutrient concentrations represent a crucial issue. If released without control into soils or water bodies, SCW can cause severe environmental impacts. Excess nitrogen and phosphorus may trigger eutrophication phenomena and significant alterations in the aquatic ecosystem [4]. Traditional reuse options include feed, direct land application, or recovery of proteins and lactose. However, these approaches have only partly reduced the environmental pressure of SCW and remain economically unfeasible for small and medium-sized dairies [1,5]. This has led to a rise in innovative valorization methods aligned with circular economy principles, such as membrane separation, fermentation, and the production of bio-based materials [2,3]. Recently, biological treatment using microalgae has emerged as a promising solution. Unlike conventional physico-chemical methods, microalgal systems can simultaneously remove nutrients from SCW and produce valuable biomass. Moreover, the exploitation of microalgae is emerging as an interesting green alternative source with a low carbon dioxide (CO_2_) footprint, further enhancing their role in sustainable bioprocesses [6]. These multiple functions address two critical challenges: mitigating the environmental impact of SCW disposal and ensuring sustainable sources of renewable resources. While many studies have shown the potential of microalgae for general dairy wastewater (DWW) treatment, specific research on SCW remains limited.

SCW should be regarded as a distinct stream within DWW. Its unique composition makes it particularly suitable for reuse and valorization, in line with circular economy strategies and global sustainability goals. This review, therefore, provides a comprehensive overview of SCW valorization through microalgae treatments, highlighting current advances, critical challenges, and future perspectives. By comparing SCW to broader dairy waste streams, we aim to clarify its specific potential and limitations, and to identify research priorities for developing scalable, cost-effective, and sustainable bioprocesses.

## 2. Characteristics and Classification of Dairy Wastewaters

**Figure 1:**
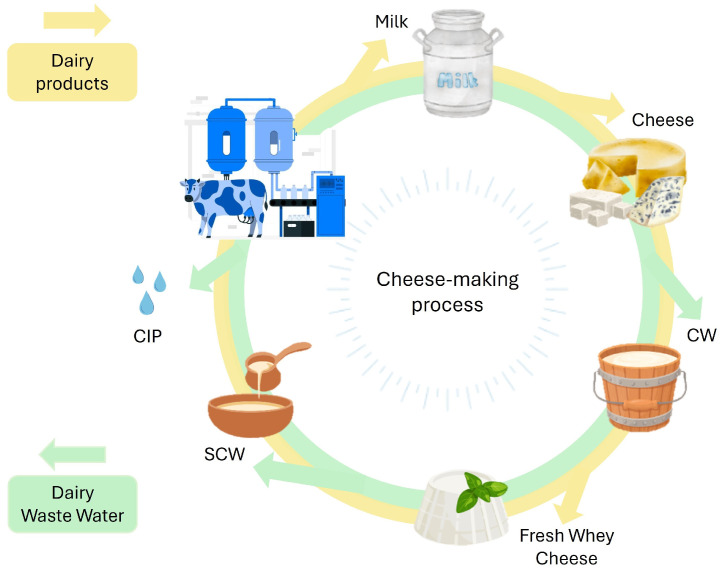
Cheese-making process and related outputs. The figure summarizes the cheese-making process from milk to final dairy products (in yellow), including cheese and fresh whey cheese. It also illustrates the generation of dairy wastewater streams (in green), such as CW, SCW, and cleaning-in-place (CIP) water.

The several DWW types (Figure 1) differ substantially in composition, physico-chemical properties, and environmental impact. Among them, the most important is cheese whey (CW), produced during the coagulation of milk proteins to form curd. Most whey worldwide comes from cow’s milk, with an estimated annual production exceeding 160 million tons [7]. CW has a yellow-green color due to riboflavin and is characterized by about 65 g L^−1^ of total solids. It represents nearly 95% of the milk volume, retaining approximately 55% of its nutrients and 20% of the proteins [5]. CW can be classified into sweet whey, obtained through enzymatic coagulation, and acid whey, generated by acid coagulation. They differ in pH, lactose, minerals, and proteins. Sweet whey typically presents pH 6–7, 46–52 g L^−1^ lactose, and 2.5–4.0 g L^−1^ minerals. In contrast, acid whey presents pH 4.5–5.8, 44–46 g L^−1^ lactose, and 4.3–7.2 g L^−1^ minerals [3]. Both the CW types represent an environmental challenge due to large volumes and high organic loads [3,5]. Approximately half of the whey produced globally is still regarded as waste, and its discharge without treatment significantly pollutes water bodies [8]. The most common conventional valorization pathway of CW is the production of fresh whey cheese through thermal aggregation, a practice still widespread in many European countries, particularly in Mediterranean regions [1,9]. Other strategies include the production of whey powder, protein concentrates, and lactose. These are widely used in the food industry for their functional properties [10,11,12], but they are often too expensive and unviable for small and medium-sized dairies [13]. The remaining liquid after the production of fresh CW represents the SCW, which still represents a considerable environmental burden due to its high lactose and mineral content. Similarly to CW, the SCW represents a significant issue for the dairy sector. A key distinction between CW and SCW lies in their protein content. CW, thanks to its residual protein fraction, can still be used to produce fresh whey cheeses. In contrast, SCW is essentially “exhausted” from a protein standpoint and cannot be used for further cheesemaking, even if it still contains residual nutrients. This lack of direct food applications makes SCW a far more problematic effluent, requiring greater attention in terms of treatment and valorization pathways. For every kilogram of fresh cheese produced, approximately 18 L of SCW are generated [14,15]. In Italy, it is estimated that around 1 million tons of SCW are produced annually, highlighting the considerable environmental and management challenges associated with its disposal. Typically, small and medium-sized companies cannot valorize the SCW components for human consumption, and this by-product is therefore primarily used as feed [5]. The composition of SCW varies widely, depending on the type of CW. SCW resulting from cow whey typically contains 0.1–0.2% proteins, 1.0–1.1% salts, and 4.8–5.0% lactose. In contrast, SCW obtained from ovine whey presents a significantly higher nutritional content, with approximately 6.7% dry matter, 0.5% protein, 0.5% fat, and 2.1% minerals [16,17,18]. In addition to CW and SCW, dairy processing also generates other wastewater streams from equipment washing, cleaning-in-place (CIP) systems, brine discharge, and accidental product losses. Depending on how DWW is managed at the facility, this mixed effluent may include residual CW, SCW, CIP water, and even municipal wastewater.

These streams are often not treated separately but are discharged together, forming a heterogeneous effluent [1]. The combination of these streams may cause a dilution effect. This can temporarily reduce pollutant concentrations and improve parameters such as pH or salinity [19,20]. However, it may also increase treatment volumes, enhance compositional variability, and introduce inhibitory compounds that interfere with downstream recovery or reuse [1,21].

## 3. Second Cheese Whey (SCW): Volumes, Challenges, and Opportunities

As previously described, SCW is the liquid effluent that remains after fresh whey cheese production. In this process, the residual proteins in CW are recovered by thermal coagulation. This process is used in the production of whey cheeses, such as ricotta (Italy), brocciu (France), requeijão (Brazil and Portugal), or urda (Eastern Europe) cheeses. During this process, CW is heated to near-boiling temperatures (85–90 °C), which causes the denaturation and aggregation of heat-sensitive whey proteins, then separated as a fresh, soft cheese. SCW appears in scientific literature under various names such as scotta, deproteinized whey, post-ricotta whey, or whey cheese whey, all of which refer to the same effluent [15,22]. Its physico-chemical composition, already discussed, is summarized in Table 1. Due to its high organic and inorganic load, SCW represents a significant environmental burden. However, considering both the volume produced and resource potential, SCW can be regarded as a promising candidate for circular and bio-based valorization strategies, provided that it is treated separately from other types of wastewaters [3,22].

### 3.1. Volumes and Geographical Distribution of SCW

**Figure 2:**
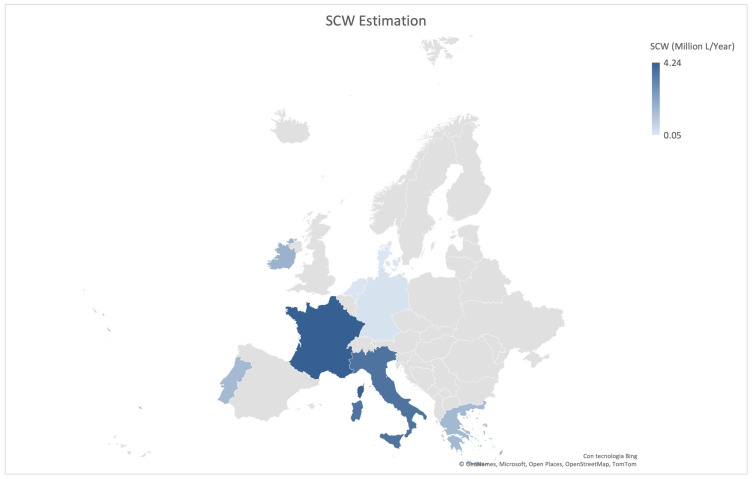
Estimated volumes of second cheese whey (SCW) generated per country. Values were obtained from ricotta export data (www.tridge.com, accessed on 1 August 2025).

Although precise global statistics on SCW production are not available, mainly due to the lack of data on CW segregation and secondary processing, some considerations can be gained through indirect estimation. In 2019, global CW production was estimated at around 158 billion liters, with the United States, India, and Brazil being the top contributors [23]. Since a portion of this volume is subjected to thermal treatment to produce soft whey cheeses, SCW is inevitably generated as a by-product. This practice is popular especially in countries with strong whey-processing traditions, such as Italy, France, Portugal, Brazil, Greece, and several Eastern European countries [22]. To approximate SCW volumes (Figure 2), national ricotta export data were used. These were combined with a standard conversion factor of 18 L of SCW per kilogram of whey cheese, as reported in the literature [3,4,15,24]. For example, in 2023, Italy exported approximately 203,380 tons of ricotta. Applying the conversion factor of 18 L of SCW per kilogram of whey cheese, this corresponds to nearly 3.7 billion liters of SCW. Based on proportional export values, France may produce around 235,000 tons of whey-based cheese, generating over 4.2 billion liters of SCW. Similar estimates suggest that Greece and Ireland could each contribute between 1.5 and 1.7 billion liters annually, while Portugal also represents a relevant source [24]. In contrast, countries such as Germany, the Netherlands, and Denmark show significantly lower ricotta export volumes. This may reflect limited production of whey cheeses and, consequently, smaller contributions to SCW generation. These amounts should be interpreted as preliminary estimates, based exclusively on ricotta export volumes. As such, they likely underestimate the actual SCW production at the national level, since they do not account for ricotta produced and consumed within internal markets. Nevertheless, they provide a useful lower-bound indication of SCW generation in countries where ricotta is an important dairy product. Even as conservative values, these estimates highlight the need for country-specific strategies for valorization and sustainable management of this underutilized by-product.

### 3.2. Environmental Impact of SCW

SCW is one of the major environmental challenges in the dairy sector. This is due to its chemical composition, its high organic load, and the large production volumes, especially in Mediterranean regions. Its untreated discharge contributes heavily to eutrophication, oxygen depletion in aquatic systems, and the disruption of natural biogeochemical cycles [3,25,26,27]. As shown by Palmieri and co-workers [28], improper disposal, such as release into municipal wastewater, can significantly increase freshwater toxicity [29]. In addition to its direct ecological burden, SCW also generates considerable indirect impacts related to handling, treatment, and transport. Life cycle assessment (LCA) studies estimate that these stages account for up to 97% of the environmental footprint associated with whey protein concentrate (WPC) production, including contributions to climate change [30,31]. Transportation may account for 28% to 70% of these impacts, especially due to the use of fossil fuels and the formation of photochemical smog. Pre-concentration of whey at the source has been shown to reduce this impact by up to 14.3%. However, even simpler valorization strategies, such as animal feed or fertilizer, do not automatically result in environmental gains. Palmieri and co-worker [28] observed that reductions in fossil fuel use did not always correspond with decreases in greenhouse gas emissions or eutrophication, particularly when productivity improvements were marginal. Similarly, conventional treatment options (aerobic, anaerobic, membrane-based) are energy-intensive, requiring 1–4 kWh m^−3^. They also generate additional downstream costs for cleaning, membrane replacement, or sludge management [32,33,34]. By contrast, integrated waste-to-energy approaches such as anaerobic co-digestion have shown substantial mitigation potential. Casallas-Ojeda and co-workers [27] reported that co-digesting CW with dairy manure reduced the carbon footprint of cheese production by up to 43.6%, lowering emissions from 5.5 to 3.1 kg CO_2_-eq per kg of cheese. In conclusion, SCW management remains a complex environmental issue. While recovery and valorization strategies can significantly reduce pollution and support circular economy goals, their true sustainability depends greatly on energy efficiency, treatment logistics, and local feasibility. Decentralized biological systems powered by solar energy are increasingly gaining interest, particularly in sun-rich regions. They offer low-footprint alternatives that align with the principles of a circular bioeconomy.

### 3.3. Industrial Applications of SCW

In this section, industrial uses specifically involving SCW are discussed, focusing on sectors where its valorization has been effectively implemented or tested at pilot scale. Table 2 reports valorization processes applied more broadly to DWW, which may serve as potential strategies for SCW in the future. One of the most developed sectors using SCW is the food industry, where it is employed for the extraction of proteins, lactose, and bioactive peptides, particularly in the production of functional foods and beverages [35]. For instance, in Ecuador, a traditional fermented whey–fruit drink known as Colada has shown antimicrobial and antioxidant properties, supporting whey’s potential as a functional ingredient [35,36]. In Europe and North America, protein-enriched beverages, such as iced green tea fortified with second whey protein (SWP) isolates, are gaining market traction [37,38]. SCW is also incorporated into commercial protein blends, although not always explicitly labeled. Brands like Optimum Nutrition and MyProtein list generic “whey protein blends,” which may include SCW [39,40]. In 2025, Arla launched the Nutrilac^®^ HighYield system in the dairy sector, integrating SCW directly into final products such as Greek yogurt, ricotta, and soft cheeses. This innovation improved nutritional value while reducing the need for waste management [41]. Lactose recovery is another key industrial use. Unlike protein blends aimed at sports or general nutrition, lactose from SCW is recovered for applications in food, pharmaceuticals, and infant nutrition. Companies such as Arla Foods, Glanbia Nutritionals have dedicated facilities for this purpose [41,42]. Membrane filtration technologies were initially designed for sweet whey, but recent advancements have allowed their application for SCW as well. However, the higher salt content and variable composition of SCW often necessitate pre-treatment or blending with other whey streams [43]. In the bioenergy sector, ethanol production from whey is well established. Microorganisms such as *Saccharomyces cerevisiae* or *Gluconobacter oxydans* can bioconvert whey into ethanol and galactonic acid. In New Zealand and the USA, for example, Anchor Ethanol Ltd. produces about 8 million gallons of ethanol annually using enzymatic hydrolysis and specialized yeast fermentation [44,45]. However, SCW’s composition makes large-scale application more difficult, and its use remains limited to pilot-scale trials [3,46,47]. Another emerging application is bioplastic production, especially polyhydroxyalkanoates (PHAs). They are produced by specific bacteria, which, under stress conditions, accumulate PHAs inside the cell for energy and carbon storage purposes. Despite its potential, SCW-based PHA production is constrained by low yields, high downstream costs, and the need for process optimization [46]. In agriculture, SCW is reused as liquid fertilizer or livestock supplement, especially in small-scale farms with limited access to synthetic inputs. Its nitrogen, phosphorus, and potassium content can improve soil fertility and forage productivity [1,3]. On-farm reuse is common in Southern Italy and Portugal [22,43]. However, without proper dilution and nutrient management, this practice can lead to eutrophication, oxygen depletion, or soil imbalance [28]. Use in livestock feeding is more limited due to hygiene concerns and variable composition, particularly in industrial settings. Furthermore, environmental assessments have shown that agricultural reuse does not always result in a lower carbon footprint. Finally, biotechnological applications are gaining attention. Thanks to its rich nutrient profile, SCW has been tested as a growth medium for probiotics, lactic acid bacteria, and microalgae [26]. Among these, microalgae cultivation is especially promising due to its capacity to utilize natural sunlight, particularly in Mediterranean regions. Additionally, it can incorporate organic substrates in mixotrophic growth systems. This combination of resource recovery and renewable energy use supports the general objectives of the circular economy, enhances biomass productivity, and reduces waste.

## 4. Microalgae for SCW Valorization: Concepts, Technologies, and Metabolisms

### 4.1. Definitions and Applications

Microalgae are unicellular photosynthetic microorganisms found in both freshwater and marine environments. They include eukaryotic taxa such as *Chlorella*, *Scenedesmus*, *Dunaliella*, as well as cyanobacteria such as *Arthrospira*, *Anabaena*, or *Synechococcus*. Like plants, they can convert sunlight, CO_2_, and inorganic nutrients into biomass rich in lipids, proteins, carbohydrates, and pigments [49]. Their simple structure, rapid growth, and ability to grow on unconventional substrates, like wastewater, make them increasingly attractive as a sustainable solution for circular bioeconomy strategies [4]. One of the most valuable features of certain microalgae, such as *Arthrospira*, *Chlorella*, and *Scenedesmus*, is their high protein content. In fact, the protein levels in these species can exceed 50–60% of their dry weight, making them comparable to or even superior to conventional protein sources, such as soybeans or meat [50,51]. These proteins are highly digestible and rich in essential amino acids, making them suitable for both human and animal nutrition [52]. Thanks to their metabolic flexibility, many strains can grow autotrophically, heterotrophically, or mixotrophically, assimilating both inorganic and organic carbon, an advantage in wastewater-based cultivation [53,54,55]. Their ability to remove nitrogen, phosphorus, and organics has promoted their use in phycoremediation systems, especially the high rate algal ponds (HRAPs) and bacterial–algal consortia [56,57]. In energy applications, microalgae can accumulate up to 50% of their dry weight as lipids under stress conditions. Saturated and monounsaturated fatty acids are used for biodiesel and biogas. By contrast, polyunsaturated fatty acids (PUFA), such as eicosapentaenoic acid (EPA) or docosahexaenoic acid (DHA) from *Nannochloropsis* or *Schizochytrium*, are highly valued in nutraceuticals and food supplements [58,59,60]. Finally, species belonging to the *Spirulina* and *Haematococcus* genera are commercialized for pigments (astaxanthin, phycocyanin), while algal biomass is explored for bioplastics, bioactive polysaccharides, and ingredients for pharma and cosmetic industries [50,53,61,62,63]. Overall, microalgae offer a versatile, low-impact platform for integrating waste valorization, nutrient recovery, and high-value bioproducts [58,62].

### 4.2. Microalgal Cultivation Systems

Microalgae cultivation can be classified into open and closed systems (photobioreactors), each with its own advantages and limitations. Open pond systems, including circular and channel ponds, are the most traditional and cost-effective methods for large-scale biomass production. They offer benefits such as low construction and operational costs, simple design, and ease of scalability [4,63,64]. These systems have significant drawbacks. They are susceptible to contamination, lose water through evaporation, provide inadequate control of environmental conditions, and generally show low biomass productivity [4,65,66]. Among open systems, raceway ponds are considered the most efficient. They use paddle wheels to ensure homogeneous mixing and homogeneous distribution of nutrients [63,67]. Their success is demonstrated by facilities like the Columbus Algal Biomass Farm, which achieved the commercial production of over 500 tons of microalgal biomass without significant technical issues [63,68]. To overcome the limitations of open systems, photobioreactors (PBRs) have emerged as closed and controlled cultivation platforms that improve biomass productivity and reduce the risk of contamination [4,63,64,69]. These systems, which include tubular, flat plate, and vertical column configurations, enable precise control over light, gas exchange, and nutrient delivery. Tubular photobioreactors (PBRs) are commonly used in industrial settings in countries like Germany and Israel for the cultivation of high-value species, belonging to *Haematococcus* and *Chlorella* genera. However, they may face limitations related to mass transfer due to the long length of the tubes [70,71]. Vertical column PBRs, which use diffusers to improve gas–liquid exchange, offer simplicity and efficiency, albeit with limited light penetration [63,72]. Flat plate PBRs maximize light exposure and minimize oxygen buildup, resulting in superior stress due to aeration and maintenance challenges that can arise on a large scale [63,73,74]. In summary, choosing a cultivation system requires balancing economic feasibility, productivity, operational complexity, and intended application. The decision also depends on geographical conditions, the desired product type, and the specific microalgal strain used.

### 4.3. Microalgal Photoautotrophy, Heterotrophy, and Mixotrophy

**Figure 3:**
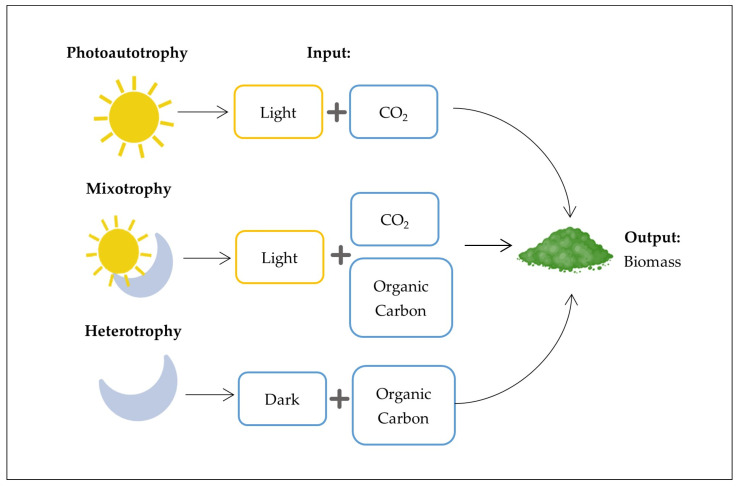
The three main metabolic pathways of microalgal trophic growth: photoautotrophic (light and inorganic carbon), mixotrophic (light with both inorganic and organic carbon), and heterotrophic (organic carbon in the absence of light).

Microalgae are versatile microorganisms that can adapt to various environmental conditions. Their adaptability comes from a flexible metabolism (Figure 3). This metabolism can switch between photoautotrophy, heterotrophy, mixotrophy, and, less commonly, photoheterotrophy. Each mode has distinct nutrient requirements, metabolic pathways, and cultivation implications [75].

Among these, photoautotrophy is the dominant trophic mode in natural ecosystems, relying on light and CO_2_ as energy and carbon sources. It is energetically favorable and environmentally beneficial due to its CO_2_-fixing ability, making it especially attractive for large-scale applications such as biofuel production and pigment extraction [76,77]. In contrast, heterotrophic cultivation operates in the absence of light, using organic carbon sources such as glucose or glycerol. This mode promotes high cell density and lipid accumulation [78], facilitating simpler reactor design. However, it also raises costs and increases the risk of microbial contamination [79]. Strains of *Chlorella vulgaris*, *Tetraselmis chuii*, and *Schizochytrium limacinum* have demonstrated successful heterotrophic growth [80]. An increasingly studied strategy is mixotrophy, which combines light-driven photosynthesis with the assimilation of organic carbon. This dual mechanism often leads to enhanced biomass and metabolite yields, while reducing CO_2_ emissions compared to pure heterotrophy [81,82]. It also preserves pigments and carotenoids, such as β-carotene, under illuminated conditions [55,83]. These characteristics make mixotrophy particularly appealing for applications involving nutrient-rich wastewaters, like agro-industrial effluents, especially in regions with abundant sunlight, where solar energy can be harnessed sustainably. Another, less common option is photoheterotrophy, which utilizes both light and organic carbon, but does not use CO_2_ as a carbon source. Despite its metabolic flexibility, this strategy is rarely applied at an industrial scale due to its limited scope and complexity [84]. The choice of trophic mode is not isolated: it depends on multiple interacting factors, including the composition of the input substrate, climatic conditions, and the intended end use of the biomass. When dealing with contaminated or nutrient-rich wastewaters, cultivation strategies typically focus on waste treatment and bioenergy recovery. In contrast, for high-value markets such as nutraceuticals or cosmetics, safety and purity are the primary concerns. The geographical context is crucial: photoautotrophic and mixotrophic cultivations are preferred in sunny regions, such as the Mediterranean basin, whereas heterotrophic cultivations may be more suitable for areas with limited light [45]. In the context of SCW valorization, mixotrophic cultivation stands out as an especially viable option, particularly in Southern Europe, where high solar irradiance coincides with increased SCW production. This method allows for the simultaneous utilization of sunlight and the organic content in SCW, aligning effectively with circular bioeconomy models focused on waste reduction and resource recovery [85,86]. Moreover, the metabolic plasticity of microalgae enables the production of a wide spectrum of bioactive compounds, including lipids, proteins, carbohydrates, pigments, vitamins, and polyunsaturated fatty acids, which supports their use as biotechnological platforms [87,88]. However, large-scale mixotrophic cultivation still faces challenges related to contamination control, process efficiency, biomass harvesting, and downstream processing [4,75,88,89]. Crucially, the trophic mode and the cultivation system are interdependent. Photoautotrophic cultures can be grown in open systems. By contrast, mixotrophy requires closed photobioreactors to prevent microbial contamination from added organic carbon sources, which may promote the growth of unwanted bacteria, yeast, or molds [90]. Therefore, the optimal configuration, both biological and technical, must be tailored to the substrate, target product, and regional context. This system-level approach is essential for designing scalable, cost-effective, and sustainable microalgae-based processes [63,91]. Table 3 provides a summary of the main characteristics of microalgal cultivation modes, comparing photoautotrophic, heterotrophic, and mixotrophic cultivation strategies based on species used, carbon source, light dependency, advantages, limitations, and key industrial applications.

## 5. Microalgal Valorization of SCW: Case Studies and Regulatory Insights

### 5.1. Valorization of Dairy Wastewater (DWW) Through Microalgal Cultivation

Although this review focuses specifically on SCW, it is relevant to consider the broader category of DWW for several reasons. First, SCW is chemically and procedurally a subset of DWW and is often generated within the same processing facilities. Second, while SCW-specific studies are still limited, extensive research has been conducted on DWW treatment using microalgae. Discussing these studies can therefore provide valuable insights and methodological references for developing targeted SCW treatment strategies. SCW has some compositional differences, such as higher organic matter content and lower dilution. However, both SWC and DWW present similar environmental challenges and offer comparable valorization opportunities.

The use of microalgae for DWW treatment has advanced significantly, with mixotrophy emerging as a dominant strategy due to its enhanced biomass productivity and effective nutrient uptake. However, recent studies indicate significant variability in operational setups, including cultivation modes, microalgal species, and integration with other technologies. Several studies employed pure mixotrophic cultivation using *Chlorella* spp., *Scenedesmus* spp., *Tetraselmis* spp., or *Galdieria* spp. with CW or diluted DWW. For instance, *Chlorella* spp. grown in 40% pretreated CW reached 0.5 g L^−1^ d^−1^ of biomass and 0.2 g L^−1^ d^−1^ of lipids, with near-complete nutrient removal [86]. Similarly, *T. chuii* achieved 98% phosphorus and 93% nitrogen removal, highlighting how light intensity and organic substrates affect productivity [80]. To reduce costs, some studies explored cultivation under non-sterile conditions. *Chlorella* spp. cultivated in untreated DWW at various dilutions (5×, 10×, 20×) produced up to 0.9 g L^−1^ in 6 days, with >84% COD removal, despite slightly reduced yields compared to sterile systems [92]. Beyond remediation, *Dunaliella tertiolecta* grown on CW showed a 2.3-fold biomass increase and 12-fold chlorophyll enhancement under mixotrophy, supporting pigment and extracellular polymeric substance (EPS) production alongside wastewater treatment [93]. While *Chlorella* spp. and *Scenedesmus* spp. are often used for lipid accumulation and biofuel applications, species like *Dunaliella* enable access to biopolymers and pigments. Integrated and consortia-based strategies are also gaining attraction. *Scenedesmus quadricauda* and *Tetraselmis suecica* used sequentially achieved 92% nitrogen and 100% phosphate removal over multiple cultivation cycles [94]. *Scenedesmus* spp. also demonstrated autotrophic–heterotrophic synergy, contributing to a 20% improvement in phosphate recovery through pathway interaction [95]. In more complex configurations, anaerobic digestion (AD) effluent was used to cultivate *Chlorella pyrenoidosa*, capturing residual nutrients post-biogas production. Biomass remained high (~0.4 g L^−1^ d^−1^), though dilution was needed to mitigate ammonium toxicity [96]. Phycoremediation–bioenergy integration was explored by Brar and co-workers [97], who tested *C. pyrenoidosa*, *Anabaena ambigua*, and *Scenedesmus abundans* in diluted DWW. Biomass yields ranged from 18–24 mg L^−1^ d^−1^, with lipid content up to 17% DW and BOD/COD reductions of 56–77%, supporting potential for methane or biodiesel production. Notably, *S. abundans*, with higher protein content, showed greater methane yield potential, underscoring how biochemical profiles shape valorization routes. Co-cultivation approaches, such as those proposed by Gupta and colleagues [54], combined filamentous and high-value microalgae to improve flocculation and system stability. Monocultures of *C. vulgaris* [98] produced about 0.6 g L^−1^ of biomass and were effective in removing 85–95% of nitrogen. However, co-cultures demonstrated greater operational robustness, although they required more complex downstream processing for lipid extraction. Additionally, the system design plays a crucial role in overall efficiency. Costa and co-workers [99] found that open ponds under mixotrophy yielded 2.6× more biomass than autotrophy alone, emphasizing how environmental and engineering factors amplify or limit biological performance [100]. Recently, several studies have explored the cultivation of *Galdieria sulphuraria* on agro-industrial wastes. For example, Sloth et al. [101] tested growth on food waste from restaurants and bakeries, while Rahman et al. [102] used granular starch from potatoes. Zimermann et al. [93] evaluated whey permeate as a carbon source, and Occhipinti et al. [55] studied mixotrophic growth on buttermilk. In the latter case, a 40% *v*/*v* of buttermilk (corresponding to 2 g L^−1^) gave the highest biomass production. The highest biomass productivity was obtained in mixotrophy (0.6 g L^−1^ d^−1^), corresponding to a carbon removal of 61%, confirming that lactose-containing substrates hold promise as a substrate for *G. sulphuraria* growth. This confirms that substrates containing lactose have potential for promoting the growth of *G. sulphuraria*. Overall, these findings suggest that there is no single superior method for cultivation; optimal outcomes depend on the alignment of microalgal traits, wastewater characteristics, and specific process goals. Lipid-rich species like *Scenedesmus* spp. and *Chlorella* spp. are suited for biofuel production, while robust strains belonging to *Tetraselmis* spp. or filamentous consortia are suitable for high-load, non-sterile systems. Integrated approaches, such as AD coupling or multi-phase phycoremediation, maximize circularity but require careful design to avoid toxicity or dilution penalties. Future developments should focus on system-specific configurations, supported by LCA analysis and techno-economic assessments, to transition microalgae-based DWW treatment from experimental setups to industrial-scale applications.

### 5.2. Microalgal Cultivation on SCW: Current Studies

Exploring the literature on DWW treatment, this section focuses specifically on SCW. Although SCW and DWW share common challenges, SCW typically presents a more concentrated profile, with elevated levels of organic compounds and total suspended solids (TSS), which affect turbidity. These characteristics require tailored microalgal cultivation strategies, including species selection, trophic mode, SCW pre-treatment, and reactor design. Despite its environmental significance, SCW is still underexplored in microalgal biotechnology. In Table 4, recent findings are shown, with limitations, and implications for future research. SCW has gained attention, mainly for its high lactose, nitrogen, and salt content. Among the proposed strategies, mixotrophic cultivation has emerged as particularly promising, enabling efficient biomass production while treating nutrient-rich effluents. This approach mitigates the limitations associated with turbidity, especially when combined with partial filtration or dilution of the substrate. *C. vulgaris* is the most exploited species in this context. For example, Casá and co-workers [103] cultivated *C. vulgaris* in diluted SCW, under mixotrophic conditions, achieving 1.6 g L^−1^ biomass with protein content up to 46% of dry weight. However, supplementation with nitrogen and phosphate combined with SCW sterilization was necessary to maintain culture stability and reduce microbial competition. Similarly, Ribeiro and co-workers [104] used *C. protothecoides* to enhance both biomass and pigment production (e.g., lutein, β-carotene), finding higher yield when SCW was combined with a basal medium. Also in this case, nutrient supplementation and sterilization were crucial for success, suggesting that SCW, as it is, is often insufficient for sustaining algal growth. *Scenedesmus acutus*, tested by Giovanardi and co-workers [105], produced lower biomass (0.9 g L^−1^) but accumulated valuable lipids and pigments during nitrogen starvation. The two-phase cultivation method, which involves initial growth followed by exposure to stress, has proven effective in increasing metabolite content. However, it requires similar levels of dilution and supplementation. A notable exception to this approach is to use *G. sulphuraria*, a red polyextremophilic microalga that can grow at temperatures up to 56 °C and in environments with a pH ranging from 0.5 to 4.0. Additionally, *G. sulphuraria* presents a versatile metabolism, being able to grow autotrophically, heterotrophically, or mixotrophically. Russo and co-workers [106] demonstrated its ability to grow on undiluted, unsterilized SCW without pH adjustment, achieving 1.8 g L^−1^ biomass. This eliminates many pre-treatment needs but restricts application to species with similar extremophilic traits and requires strict pH control (<3) to prevent contamination. Across studies, three recurring issues emerge: the need for SCW dilution, nutrient supplementation, and contamination control. Due to SCW’s turbidity and high BOD, photoautotrophic cultivation is generally unfeasible, leading most studies to rely on mixotrophy or heterotrophy. While these modes leverage SCW’s organic carbon, they also allow microbial contamination, especially in open systems, necessitating sterilization or pasteurization, which raises operational costs. Reported biomass yields vary (0.9–3.6 g L^−1^), with protein contents of 22–46% of dry weight and lipids up to 20% of dry weight. The biochemical diversity of microalgal biomass grown on SCW supports applications across feed, nutraceuticals, bioenergy, and pigments. Moreover, *G. sulphuraria* can produce valuable pigments such as C-phycocyanin (C-PC), allophycocyanin, and chlorophyll. These compounds are used as dyes in diagnostic histochemistry, as colorants in cosmetics and the food industry, and as antioxidants in the pharmaceutical sector. Currently, commercially, C-PC is produced by *Spirulina (Arthrospira) platensis* in phototrophic cultures. Its production on a large scale, in mixotrophy mode, using *G. sulphuraria*, appears to be an interesting strategy. Occhipinti and co-workers [55] found that the C-C-PC content in *G. sulphuraria* grown in lactose-based medium was comparable to that obtained in glucose-limited conditions. This finding was discussed as due to the lower lactose uptake of *G. sulphuraria*, resulting in a lower growth rate and a higher C-PC content. However, challenges persist in achieving industrial scalability, particularly regarding light distribution, cost-effective harvesting, and reducing reliance on sterile conditions [4,55]. *Chlorella* spp. and *Scenedesmus* spp. produce good yields but need clean, supplemented media. Meanwhile, *G. sulphuraria* minimizes the need for pre-treatment but is constrained by its specific habitat requirements [107]. Innovative directions include microbial consortia, which may enhance lactose breakdown and contamination resistance [108], or bioreactor designs tailored to low-input systems.

**Figure 4:**
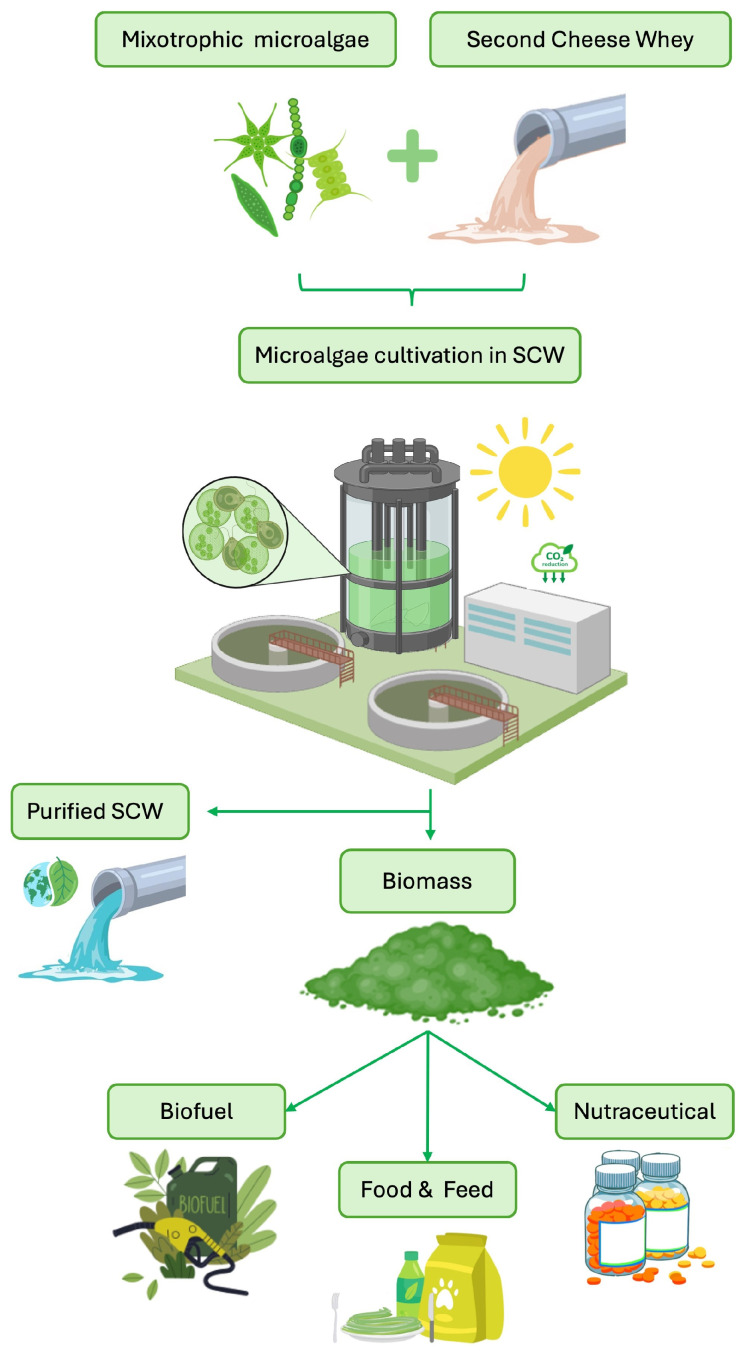
Biorefinery concept of SCW valorization through microalgae cultivation. The process enables SCW bioremediation and the production of biomass, which can be directed to various applications, including biofuels, food and feed, and nutraceuticals.

The choice of strain, trophic mode, and process design should match the product goal and environmental context. The strategy must balance productivity with both economic and ecological sustainability. There are many variables to consider and, depending on them, the outputs may differ. The microalgal biomass obtained from SCW can in fact be directed to several application sectors, including bioenergy, fertilizers and biostimulants, aquaculture, feed, and potentially nutraceuticals and cosmetics. In addition to these uses, SCW-derived biomass can also provide value-added compounds like carotenoids or phycobiliproteins [49,93,105], PUFAs [61,62], proteins [51,52,53,54,55,56,57,103], and bioactive polysaccharides [50,51,52,53,57,61,62,63], supporting applications in nutraceutical, cosmetic, feed, and pharmaceutical markets (Figure 4). However, not all these options are immediately accessible, as the use of biomass is subject to strict regulations that affect its practical feasibility and market entry. The following section, therefore, analyzes the main legislative frameworks, with particular attention to the limits and opportunities they pose for the reuse of treated SCW and the derived biomass.

### 5.3. Microalgal Biomass from Treated SCW: Efficiency and Regulation

SCW, due to its high organic and nutrient load, serves as an effective substrate for microalgal cultivation. This organic richness supports high biomass productivity and enables a parallel process of wastewater treatment, as microalgae assimilate nitrogen, phosphorus, and carbon compounds during growth. Numerous studies have shown that microalgal systems operating on SCW and other agro-industrial wastewaters (AWWs) can achieve substantial pollutant removal. For instance, PBRs and HRAPs applied to dairy effluents have reached COD and total phosphorus removal rates of 50–100%, with ammonium being fully removed under optimized conditions [109,110]. BOD reductions above 80% have been documented in SCW-specific setups [111], highlighting the strong depurative capacity of this approach. Hybrid strategies combining algae with fungi or bacteria have further improved performance, with fungi–microalgae consortia achieving COD removal up to 70% and NH_4_-N over 94% [112] and bacterial co-cultures enabling nitrogen removal above 80% and phosphorus up to 70% [113]. Compared to conventional treatments, microalgal systems offer added benefits such as low energy inputs, reduced sludge production, and biomass recovery for valorization [114,115,116]. Despite the strong technical feasibility of microalgal cultivation using SCW, which can effectively remediate wastewater while producing high-value biomass, significant regulatory challenges to its practical implementation persist. Specifically, the treated effluent must comply with the discharge limits set by the Urban Wastewater Treatment Directive [117]. For agricultural reuse, it must also meet the requirements of the more recent European Regulation [118], which defines minimum quality standards and risk management rules for irrigation water. These include not only physicochemical parameters, such as BOD_5_ < 25 mg L^−1^, COD < 125 mg L^−1^, total nitrogen < 15–20 mg L^−1^, according to the person equivalent (PE), and total phosphorus <2 mg L^−1^. In addition to these parameters, there are specific microbiological criteria, particularly regarding *Escherichia coli*, where acceptable concentrations vary based on the intended agricultural use of the water. Furthermore, the valorization of the resulting biomass is equally subjected to strict legal constraints, as reported in Table 5. When SCW is used as a culture medium, the produced algal biomass is considered waste-derived, and its reuse is permitted only under certain conditions, depending on the final application. Bioenergy production (e.g., biogas, biodiesel, bioethanol) remains the most viable and least restricted route, as non-food applications are not subject to the same safety standards [119,120]. The use of algal biomass as fertilizer or biostimulant is permitted under European Regulation [121], but it must meet strict limits on heavy metals, pathogens, and persistent organic pollutants [122]. When used in feed, it must comply with European legislation on feed marketing [123] and on undesirable substances in feed [124], which establishes requirements for traceability and sets thresholds for contaminants, including mycotoxins and heavy metals. Even when established microalgae recognized for their favorable nutritional profiles, such as *C. vulgaris*, are grown on whey, microbial contamination and endotoxins remain critical safety concerns [125]. The greatest regulatory barriers apply to human-related markets, including nutraceuticals, cosmetics, and functional foods. In such cases, the European Novel Food Regulation [126] and the Cosmetics Regulation [127] restrict the use of biomass from non-approved sources or waste-based substrates, unless extensive purification and toxicological validation are performed [122,125]. Consequently, biomass derived from SCW is generally excluded from these high-value sectors unless processed to pharmaceutical-grade standards. In summary, SCW valorization through microalgal cultivation is technically robust and environmentally sustainable, but its industrial adoption is still limited by fragmented and restrictive regulations. Progress will depend on process optimization, cost reduction, and clearer regulatory pathways for waste-derived resources. To move from proof-of-concept to large-scale use, cultivation strategies must be aligned with viable end uses (such as bioenergy, agriculture, feed) while ensuring economic and ecological sustainability [119,122,125].

### 5.4. Economic Feasibility and LCA of SCW Valorization

The literature does not provide harmonized cost estimates specific to SCW (€/kg biomass or €/m^3^ treated), but solid indications come from real microalgae production facilities. In a demonstrative tubular photobioreactor setup, Acién et al. [90] reported a production cost of approximately 69 €/kg of dry biomass. Scale-up scenarios of 200 metric tons annually, with energy optimization and waste stream utilization, reduce the potential cost to 12.6 €/kg. Other studies demonstrate that using agro-industrial wastewater or anaerobic digestion effluents can significantly cut operating costs by replacing fertilizers and utilities. Recent estimates indicate values between 2 and 4 €/kg in optimized semi-industrial systems [96,97], and even less than 1 €/kg in large-scale scenarios with non-sterile cultivation and high productivity levels [98]. When applied to SCW, utilizing this dairy by-product as a mixotrophic substrate decreases the need for expensive pre-treatments (such as sterilization or pH control, nutrient addition) and boosts productivity in open ponds—up to 2.6 times higher than autotrophy [99,100]. Integration with AD provides further benefits by supplying CO_2_ and nutrients, while also enabling the use of algal biomass for bio-energy products [96,97,98].

On the environmental side, LCA studies show that cultivation on agri-food wastewaters can reduce impacts up to 5-fold compared to synthetic media, mainly due to lower chemical demand and the “co-treatment effect” of nutrient/organic removal [128,129]. In a comparison between conventional wastewater treatment plants (WWTPs) and WWTPs integrated with microalgal ponds, the latter improve 14 out of 16 impact categories when microalgal biomass is used as a bio-stimulant, thanks to the substitution of synthetic fertilizers [130]. Furthermore, LCA studies of algal systems cultivated on wastewater report mitigations of 1.7–2.1 kg CO_2_-eq per kg biomass when replacing commercial media with wastewaters at equal yields [131]. Applied to SCW, these findings suggest measurable reductions in global warming potential (GWP, kg CO_2_-eq), eutrophication (kg PO_4_-eq/N-eq), and fossil energy demand, particularly when combining high productivity, minimized pre-treatments (e.g., acidophilic strains), and co-integration with AD and co-location at dairies [94,95,96,97,99,100,106].

## 6. Future Prospects

This study has highlighted both the challenges and opportunities related to SCW, a by-product of the dairy industry that remains largely overlooked within a circular framework. Although it is sometimes included in the broader group of DWW, this assimilation has clear limitations. SCW presents specific compositional and management features that justify a dedicated analysis. Such ambiguity in classification has likely contributed to the scarcity of studies focused on this effluent, particularly in the field of microalgal-based processes, which appear among the most promising research areas.

To address these gaps, it is crucial to develop reliable monitoring tools and shared analytical models able to quantify directly and precisely the volumes generated and their impact. Likewise, LCA studies are needed to define more accurately the sustainability and the environmental and economic benefits of microalgal utilization. This approach will help clarify the role of SCW within dairy waste streams and support the design of effective valorization strategies. In this context, future regulations should also evolve towards clearer and less restrictive frameworks for the reuse of microalgal biomass derived from by-products, facilitating safe and sustainable application.

## 7. Conclusions

The SCW is an underutilized by-product of the dairy industry, despite its high nutritional value and significant environmental impact. Cultivating microalgae, especially under mixotrophic conditions, offers a valuable opportunity to treat this by-product and produce valuable biomass. The transition from successful laboratory experiments to industrial implementation faces several challenges, including process optimization, cost efficiency, and regulatory compliance. Current European regulations primarily restrict the reuse of biomass derived from waste-based substrates in food and feed applications, limiting access to high-value markets. However, there are more accessible opportunities in bioenergy and agriculture, especially when accompanied by purification processes and risk assessment protocols. To fully harness the potential of SCW within a circular bioeconomy framework, future efforts must focus not only on technological innovation but also on addressing regulatory and market alignment.

## Figures and Tables

**Table 1:** Physico-chemical properties of different Second Cheese Whey types.

Type of SCW	pH	COD (g/L)	BOD (g/L)	Lactose (%)	Proteins (%)	Salts (%)	Dry Matter (%)	Notes	References
From cow’s milk	~6.0	~60	~45	4.8–5.0	0.1–0.2	1.0–1.1	~6.0	Average data from small dairies	[14]
From sheep’s milk	6.2–6.5	n.d.	n.d.	4.5–5.0	~0.5	~0.5	~6.7	Richer composition compared to cow SCW	[18]
From mixed milk	6.0–6.5	50–70	n.d.	4.7–5.2	0.2–0.3	0.9–1.1	~6.5	Variability linked to the type of milk	[17]
From cow’s milk	~6.1	n.d.	n.d.	~5.0	~0.2	~1.1	~6.0	Brazilian study	[16]

n.d.: note determined.

**Table 2:** Valorization strategies for second cheese whey (SCW): Applications, strengths, and industrial potential.

Valorization Strategy	End-Product Applications	Opportunities/Strengths	Challenges/Limits	Industrial Perspective	References
Anaerobic digestion	Biogas, renewable energy	Established technology; renewable energy incentives; reduces GHG emissions	High capital cost; requires skilled operation	Already applied in the agro-industry	[27,47]
Bioethanol & galactonic acid production	Biofuels, chemical intermediates	Renewable alternative fuels; valorize lactose	Process optimization needed; market competitiveness	Pilot-scale; potential in biorefineries	[48]
Lactic acid fermentation	Bioplastics, food additives	Bio-based alternative chemicals	Process optimization; market competition	Early-stage research	[3]
Microalgae cultivation	Algal biomass (nutraceuticals, biofuels)	Combines treatment & biomass production; sunlight-driven	Scalability; contamination risks; harvesting	Research stage: promising for low-impact valorization	[26,46]
Microbial fermentation substrate	Enzymes, bioactive compounds	Supports microbial growth; biotechnological interest	Process standardization; economic viability	Applied in niche biotech sectors	[3]
PHA (bioplastic) production	Biodegradable plastics	Circular bio-based product; reduces fossil plastic dependency	Low yields; high production & extraction costs	Pilot-scale; emerging market	[46]
Prebiotic oligosaccharide synthesis	Functional food ingredients	Nutraceutical interest: high-value products	Complex purification; limited demand	Niche applications; limited scale	[43]
Probiotic bacteria substrate	Starter cultures, probiotics	Sustainable bio-production; waste valorization	Composition variability; small-scale use	Applied in dairy industries; niche applications	[3]
Refined lactose extraction	Food and pharma ingredient	High lactose recovery; ingredient market	Costly separation technologies	Industrial in large plants; costly for SMEs	[43]
Succinic acid production	Biopolymers, green chemicals	Circular chemical production; green chemistry	Low yields; strain development	Research stage: promising for green industries	[3]

**Table 3:** Microalgal cultivation modes and their characteristics.

Metabolism	Species	Light & Carbon Source	Productivity (g L^−1^ d^−1^)	Recovery/Waste Utilization Rate (%)	Limitations/Costs	Applications	References
Photoautotrophic	*Chlorella* sp., *Dunaliella salina*	Light (natural or artificial) + CO_2_ (inorganic)	~0.1–0.2	N and P removal 80–95%; COD/BOD reduction 60–85%	Low input costs (light, CO_2_), but low productivity makes biomass more expensive; downstream processing accounts for 50–70% of total costs	Biofuels, CO_2_ mitigation, wastewater treatment	[56,76,89]
Heterotrophic	*Chlorella protothecoides*, *Crypthecodinium cohnii*	No light + Glucose, acetate, glycerol (organic carbon)	~0.3–1.5	N removal 85–93%; COD removal 65–80%	High costs due to sterile conditions and organic substrates; using agri-food wastewater as carbon source reduces carbon cost by up to 70% compared to glucose	Nutraceuticals, lipids, industrial pigments	[78,80]
Mixotrophic	*C. vulgaris*, *Tetraselmis* sp., *Nannochloropsis salina*	Light (natural or artificial) + CO_2_ + Glucose (organic carbon)	~0.4–1.8	COD 84–90%; N 80–95%; P 90–98%	Intermediate costs; economic advantage from coupling wastewater treatment with biomass production; carbon source costs reduced by 60–70% when using agri-food wastewater	Integrated biorefineries, SCW valorization, high-value compounds	[23,81,82]

**Table 4:** Microalgal species cultivated on second cheese whey (SCW) and their trophic mode, treatment requirements, biomass productivity, key metabolites, strengths, and weaknesses.

Microalgae	Trophic Mode	SCW Treatment	Biomass Yield (g/L)	Key Metabolites	Strengths	Weaknesses	References
*C. vulgaris*	Mixotrophic	Dilution, pH adjustment, nutrient supplementation	1.6	Proteins	Good growth protein accumulation	Requires pH and nutrient adjustment	[103]
*C. protothecoides*	Mixotrophic	Autoclaving, filtration, dilution, nutrient supplementation	3.6	Chlorophyll, β-carotene, lutein	High pigment yield, effective nutrient use	Requires sterilization, sensitive to contamination	[104]
*S. acutus*	Mixotrophic	Dilution, nutrient supplementation	0.9	Lipids, pigments	High pigment production, improved lipids under starvation	Requires dilution and nutrient supplementation, lower yield	[105]
*G. sulphuraria*	Heterotrophic	None	1.8	Biomass content	Biomass rich in organic compounds, no pretreatment needed, high tolerance to acidity	Requires low pH, limited application range	[106]

**Table 5:** Main valorization routes for microalgae cultivated on second cheese whey (SCW), highlighting current European regulations, essential compliance requirements, and feasibility levels.

Valorization Pathway	Relevant Regulation	Main Requirements	Feasibility	References
Bioenergy (biodiesel, biogas, bioethanol)	No specific EU regulation	None for non-food use; quality control for emissions and residues	High, widely feasible	[119,120]
Agricultural use (fertilizer, biostimulant)	Regulation (EU) 2019/1009	Limits on heavy metals, pathogens, and persistent organic pollutants	Moderate, requires post-processing to meet criteria	[122]
Feed	Regulation (EC) No 767/2009 and Directive 2002/32/EC	Traceability, contaminant thresholds (e.g., heavy metals, mycotoxins), hygienic processing	Moderate to low, subject to strict monitoring	[125]
Human applications (nutraceuticals, cosmetics, food)	Regulation (EU) 2015/2283; Regulation (EC) No 1223/2009	Use of approved sources; safety validation; pharmaceutical-grade purification	Low, not allowed without advanced purification and validation	[122,125]

## Data Availability

No new data were created or analyzed in this study.

## Abbreviations

The following abbreviations are used in this manuscript:

AD	anaerobic digestion
AWW	agro-industrial wastewaters
BOD	Biochemical oxygen demand
CIP	cleaning-in-place
C-PC	C-phycocyanin
COD	chemical oxygen demand
CW	cheese whey
DHA	docosahexaenoic acid
DW	dry weight
DWW	dairy wastewater
EPA	eicosapentaenoic acid
EPS	extracellular polymeric substance
HRAP	high rate algal ponds
GHG	Greenhouse Gas
LCA	life cycle assessment
PBR	photobioreactors
PE	person equivalent
PHAs	polyhydroxyalkanoates
PUFAs	polyunsaturated fatty acids
SWP	second whey protein
SCW	second cheese whey
TSSs	total suspended solids
WPC	whey protein concentrate
